# Separated Micelles Formation of pH-Responsive Random and Block Copolymers Containing Phosphorylcholine Groups

**DOI:** 10.3390/polym14030577

**Published:** 2022-01-31

**Authors:** Thi Lien Nguyen, Kazuhiko Ishihara, Shin-ichi Yusa

**Affiliations:** 1 Department of Applied Chemistry, Graduate School of Engineering, University of Hyogo, 2167 Shosha, Himeji, Hyogo 671-2280, Japan; nguyenlienk56hh@gmail.com; 2 Department of Materials Engineering, The University of Tokyo, 7-3-1 Hongo, Bunkyo-ku, Tokyo 113-8656, Japan; ishihara@mpc.t.u-tokyo.ac.jp

**Keywords:** phosphorylcholine, pH-responsive, separated micelle

## Abstract

The self-assembly of pH-responsive random and block copolymers composed of 2-(*N,N*-diisopropylamino)ethyl methacrylate and 2-methacryloyloxyethyl phosphorylcholine was investigated in aqueous media. Their pH-responsive behaviors were investigated in aqueous media by dynamic light scattering (DLS) and fluorescence measurements using a pyrene hydrophobic fluorescence probe. In an acidic environment, these copolymers existed as single polymer chains that did not interact with each other. In contrast, upon increasing the pH of the solution above the critical value of ~8, separated micelles were formed in the mixture, which was indicated by bimodal distribution in DLS results with radius of 4.5 and 10.4 nm, corresponding to the random and block copolymer micelles, respectively. Fluorescence resonance energy transfer efficiencies were near to zero in the mixture of the donor labeled block and acceptor labeled random copolymers under both acidic and basic pH. These results demonstrated the coexistence of two distinct micelles.

## 1. Introduction

Amphiphilic polymer micelles have been reported to serve as carriers for the delivery of hydrophobic drugs to targeted tissues [1,2,3,4,5,6]. Among the various kinds of polymers, pH-responsive polymers are attractive because of their sensitivity to changes in the solution pH. 2-(*N,N*-Diisopropylamino)ethyl methacrylate (DPAEMA) is one of the most widely used compounds for producing pH-responsive polymers with an acid constant (p*K*_a_) of 6.8 [7,8,9,10]. The resulting polymer is deprotonated at physiological pH to form polymer micelles. Hence, hydrophobic guest molecules such as drugs can be encapsulated and transported into tumors, whose extracellular pH is generally acidic [11] and causes ionization of the pH-responsive polymer, resulting in the dissociation of the polymer micelles and the subsequent release of the entrapped molecules for cell treatment. Castro et al. [12] prepared a set of DPAEMA-contained diblock copolymers with three different hydrophilic blocks, including poly(2-methacryloyloxyethyl phosphorylcholine) (PMPC), poly (ethylene oxide) (PEO), and poly (*N*-(2-hydroxypropyl) methacrylamide) (PHPMA). In water, all the copolymers self-assembled to form nanoparticles with core–shell structures. Although the size of the aggregates produced from PEO_122_-*b*-DPAEMA_43_ and PHPMA_64_-*b*-PDPAEMA_72_ was smaller than that of PMPC_40_-*b*-PDPAEMA_70_, the former polymer aggregates were internalized in cancer cells to a lower extent as compared to healthy cells than the latter. These results indicated that the presence of PMPC blocks in the aggregates contributed to strengthen the binding of PMPC_40_-*b*-PDPAEMA_70_ to the cell membranes. Additionally, cell viability tests evidenced that these nanoparticles did not show any significant toxicity to telomerase-immortalized rhesus fibroblasts and HeLa cells and were, therefore, suitable for biomedical applications. In this context, copolymers containing 2-methacryloyloxyethyl phosphorylcholine (MPC) have been widely investigated as biomaterials [13,14,15,16,17]. The polymers containing a PMPC block or MPC units maintain their highly hydrophilic character over a wide range of aqueous solution conditions.

In addition to the development of polymer micelles formed from single polymers, the blending of two or more individual polymers or mixing of polymers and surfactants or drugs to construct polymer micelles systems with targeted properties conveniently has been also studied [18,19,20,21,22,23,24,25]. For instance, Atanase et al. [19] has studied the micellization of pH-stimulable poly (2-vinylpyridine)-*block*-poly (ethylene oxide) (P2VP-*b*-PEO) with different molecular weight in the presence and absence of sodium dodecylsulfate (SDS). They reported that without SDS, the micellization of P2VP-*b*-PEO occurred at a pH greater than or equal to 5. SDS induced the complex formation between it and the copolymer due to electrostatic interaction at pH 2 and 3 and low copolymer concentration where the copolymers exist as unimers. In another study, Atanase et al. [20] investigated the complex formation of pH-sensitive triblock copolymers poly (butadiene)-*block*-P2VP-*block*-PEO and SDS with constant P2VP block and increasing PEO content. After mixing the copolymers with SDS, at pH 7, the particle size decreased as an indication for the formation of the polymers/surfactant complexes by hydrophobic/hydrophilic interactions between SDS and unprotonated P2VP. At pH 3, the formation of core–shell–corona micelles was observed in the absence of SDS whereas in the presence SDS, complexes with smaller particle sizes were formed by the interaction between SDS and protonated P2VP, leading to the shrinkage of the shell. These suggested that the deprotonation/protonation, which upon pH is important to the micellization behavior of the copolymers. Additionally, Jiang et al. [26] reported that blends of random and block polymers composed of ethylene glycol and pyridyl disulfide ethyl methacrylate coassembled into micelles, whose hydrodynamic size, hydrophobic cargo loading capacity, and glutathione-trigged cargo release could be tuned by changing the composition of the polymer blends, which was important for the hydrophobic–hydrophilic balance of the mixtures. Liu et al. [23] demonstrated that the presence of a poly (styrene-*co*-methacrylic acid) random copolymer affected the assembly of polystyrene-*b*-poly (acrylic acid) block copolymers. When the random copolymer is a minor component in the mixture, vesicles are preferably formed on the interface with the random copolymer, whereas large spheres are formed with the copolymer block if the random copolymer is the main component in the blend. When the weight fraction of the random polymer is high, the polymers precipitate from the solution because the amount of the block copolymer is not sufficient to stabilize the aggregates. In contrast, Abbas et al. [27] indicated that when mixing two different polymers such as polystyrene-*b*-polyisoprene (SI) and polystyrene-*b*-polydimethylsiloxane (SD) in diethyl phthalate, a mixture of distinct SI and SD micelles was formed. These studies suggest that the compatibility between the polymers is a crucial factor for their association to form mixed or separated micelles.

**Figure 1:**
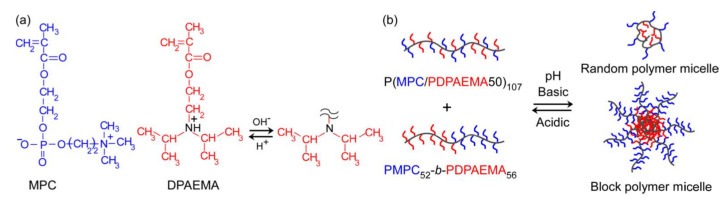
(**a**) Chemical structures of MPC and DPAEMA, and pH-responsive behavior of DPAEMA. (**b**) Conceptual illustration of the pH-induced formation of separated micelles in a mixed aqueous solution of the random and block copolymers consisting of MPC and DPAEMA.

Herein, random and block copolymers were prepared from DPAEMA and MPC monomers via a controlled polymerization method, and the pH-responsive behavior of these polymers was studied. The synthesis and behavior of the block copolymers have been reported before [12]. However, the preparation of the random copolymer and mixture behavior of these copolymers has not been discovered. Hence, the aim of this study was to investigate the formation of mixed or separated micelles in the mixture of these random and block copolymers (Figure 1). The results of dynamic light scattering (DLS) and fluorescence resonance energy transfer (FRET) analyses suggested the coexistence of two distinct micelles.

## 2. Materials and Methods

### 2.1. Materials

DPAEMA (97%) was obtained from Sigma-Aldrich (St. Louis, MO, USA) and purified using a Sigma-Aldrich inhibitor removal column. MPC was purchased from NOF Corp. (Tokyo, Japan), which was synthesized and purified following a previously reported method [28]. 4-Cyanopentanoic acid dithiobenzoate (CPD) was synthesized as a chain transfer agent (CTA) according to a reported procedure [29]. Tris(2-carboxyethyl)phosphine hydrochloride (TCEP, 98%), ethylenediamine (>99.0%), and 4,4′-azobis-(4-cyanovaleric acid) (V-501, 98%) were purchased from Wako Pure Chemical (Osaka, Japan) and used without any purification procedure. Pyrene (Py, 97%) was supplied by Wako Pure Chemical and purified via recrystallization from methanol. Alexa Fluor 555 C_2_ maleimide (A555) and Alexa Fluor 488 C_5_ maleimide (A488) were purchased from Thermo Fisher Scientific (Tokyo, Japan) and used as received. Methanol and ethanol were dried over 4Å molecular sieves and purified via distillation. Water was purified using an ion-exchange column.

### 2.2. Synthesis of a Random Copolymer, P(MPC/DPAEMA50)_107_

P(MPC/DPAEMA50)_107_ was synthesized via reversible addition−fragmentation chain-transfer (RAFT) polymerization (Scheme S1a). Briefly, MPC (3.00 g, 0.010 mol), DPAEMA (2.234 g, 0.010 mol), V-501 (22.8 mg, 0.081 mmol), and CPD (56.7 mg, 0.203 mmol) were dissolved in ethanol (20.3 mL), and the mixture was purged with argon gas for 30 min. The polymerization was conducted at 70 °C for 18 h. The reaction solution was dialyzed against ethanol for one day and then against pure water for another day. The total monomer conversion was 97.3%. The polymer was recovered by a freeze-drying method (5.0 g, 95.0%).

### 2.3. Synthesis of a Block Copolymer, PMPC_52_-b-PDPAEMA_56_

The PMPC_52_-*b*-PDPAEMA_56_ block copolymer, which was composed of PMPC and PDPAEMA blocks, was synthesized via RAFT polymerization as follows (Scheme S1b): MPC (5.00 g, 16.9 mmol), CPD (94.6 mg, 0.339 mmol), and V-501 (23.7 mg, 0.0846 mmol) were dissolved in a mixed solvent (17.0 mL) of water and methanol (4/1 *v*/*v*). The solution was transferred into a 50 mL round-bottom flask equipped with a magnetic stirrer and then degassed by purging with argon gas for 30 min. The polymerization was performed at 70 °C for 6 h. The MPC monomer conversion was 99.6%. The polymerization mixture was dialyzed against pure water for two days. The polymer was recovered by freeze-drying (4.61 g, 92.2%). The degree of polymerization (DP) and number-average molecular weight (*M*_n_) were estimated to be 52 and 1.56 × 10^4^ g/mol, respectively, on the basis of ^1^H nuclear magnetic resonance (NMR) measurements. The *M*_n_(GPC) and molecular weight distribution (*M*_w_/*M*_n_) determined by gel-permeation chromatography (GPC) were 1.41 × 10^4^ g/mol and 1.09, respectively. The obtained PMPC_52_ was used as a macro-CTA to prepare the block copolymers as follows: PMPC_52_-CTA (2.00 g, 0.128 mmol), DPAEMA (1.41 g, 6.60 mmol), and V-501 (14.5 mg, 0.0516 mmol) were dissolved in ethanol (13.0 mL). The solution was degassed by purging with argon gas for 30 min and heated at 70 °C in an oil bath for 18 h. The DPAEMA conversion was estimated to be 93.4% according to ^1^H NMR measurements. The reaction mixture was dialyzed against ethanol for one day and then against pure water for another day. After dialysis, the PMPC-*b*-PDPAEMA block copolymer was recovered by freeze-drying (3.06 g, 89.7%).

### 2.4. Fluorescence Labeling of Copolymers

The block copolymer was labeled with A488 using the protocol depicted in Scheme S2a, according to which PMPC_52_-*b*-PMPC_56_ (0.401 g) was dissolved in methanol (5 mL) and transferred into a round bottom flask. Next, 0.199 g of NaBH_4_ was dissolved in 5 mL of methanol, and the mixture was transferred to the flask of the polymer solution. After allowing the reaction to proceed at 25 °C for 2 h, the solution was dialyzed against pure water for two days. Subsequently, the copolymer was recovered by freeze-drying (0.319 g, 79.6%). The obtained copolymer (0.213 g, 0.00744 mmol) and TCEP (0.0220 g, 0.0768 mmol) were separately dissolved in 1.5 mL of a mixture of dimethyl sulfoxide (DMSO) and methanol (2/1 *v*/*v*). After that, the TCEP solution was transferred into the flask of the copolymer solution. The resulting solution was purged with argon gas for 30 min and then allowed to react for 24 h. Next, the PMPC_52_-*b*-PMPC_56_-SH solution was charged with a solution of A488 in DMSO (0.2 mL, 0.00139 mmol). A catalytic amount of ethylenediamine was added to obtain a mole ratio of ethylenediamine to polymeric thiol of 1:1, and the reaction mixture was allowed to react for 24 h at 25 °C. The solution was dialyzed against methanol for one day and then against water for two days. The labeled polymer was recovered by freeze-drying (0.142 g, 66.7%). During this process, the solution was protected from light as much as possible by wrapping the containers in aluminum foil.

The random copolymer was labeled with A555 using the same method as that described for the preparation of the A488-labeled block copolymer (Scheme S2b). As a result, A555-labeled P(MPC/PMPC50)_107_ was recovered by freeze-drying (0.183 g, 83.2%).

### 2.5. Preparation of Sample Solutions

Stock polymer solutions were prepared at a polymer concentration (*C*_p_) of 10.0 g/L as follows: The random and block copolymers were separately dissolved in 0.1 M NaCl aqueous solution at pH 2. The polymer solutions were stirred at 50 °C for one night and then stirred at room temperature for one day to ensure complete dissolution. Other solutions at desired *C*_p_ were prepared by diluting a polymer stock solution with 0.1 M NaCl. The pH of the solutions was adjusted using 1 or 6 M NaOH or HCl to obtain the desired values.

### 2.6. Measurements

^1^H NMR spectra of all the copolymers were recorded in methanol-*d*_4_ on a Jeol (Tokyo, Japan) JNM-ECZ 400 MHz spectrometer. GPC measurements were performed using chromatography systems equipped with refractive index detectors at 40 °C. Phosphate buffer (10 mM, pH 9.0) and 0.5 M aqueous acetic acid containing 0.3 M Na_2_SO_4_ solutions were used as eluents for analyzing PMPC and the copolymers, respectively, at a flow rate of 0.6 mL/min. The *M*_n_(GPC) and *M*_w_/*M*_n_ values for PMPC and the copolymers were estimated using universal curves constructed from poly (styrenesulfonate) and poly(2-vinylpyridine) standard solutions, respectively. The fluorescence spectra of the Py/copolymer and mixtures of labeled and non-labeled copolymer solutions were recorded using a Hitachi (Tokyo, Japan) F-7000 fluorescence spectrophotometer. Ultraviolet–visible (UV–vis) spectra of the labeled block copolymers were recorded using a Jasco (Tokyo, Japan) V-730 UV–vis spectrometer at 25 °C. DLS measurements were performed using a Malvern Zetasizer (Worcestershire, UK) equipped with an He–Ne laser (4 mW at 632.8 nm) at 25 °C in backscattering mode. Malvern (Worcestershire, UK) Zetasizer 7.11 software was used to analyze the obtained DLS data. Transmission electron microscopy (TEM) was performed using a Jeol (Tokyo, Japan) JEM-2100F microscope with an acceleration voltage of 160 kV. The samples were prepared by placing a drop (10 μL) of each aqueous polymer solution onto different Jeol (Tokyo, Japan) 150-mesh copper TEM grids. The excess water was blotted using filter paper, and the samples were then stained with a 0.1 wt% phosphotungstic acid aqueous solution. Subsequently, the samples were dried under vacuum at room temperature. Static light scattering (SLS) measurements were conducted using an Otsuka Electronic Photal (Osaka, Japan) DLS-7000 at 25 °C with an He–Ne laser as a light source (10.0 mW at 632.8 nm). The weight-average molecular weight (*M*_w_) and radius of gyration (*R*_g_) were estimated on the basis of Zimm plots. The values of RI increment (d*n*/d*C*_p_) were determined using an Otsuka Electronics Photal (Osaka, Japan) DRM-3000 differential refractometer at 25 °C. Sample solutions were filtered using 0.2 μm pore size membranes before performing the GPC, DLS, and SLS measurements.

## 3. Results and Discussion

### 3.1. Preparation and Characterization of Random and Block Copolymers

**Figure 2:**
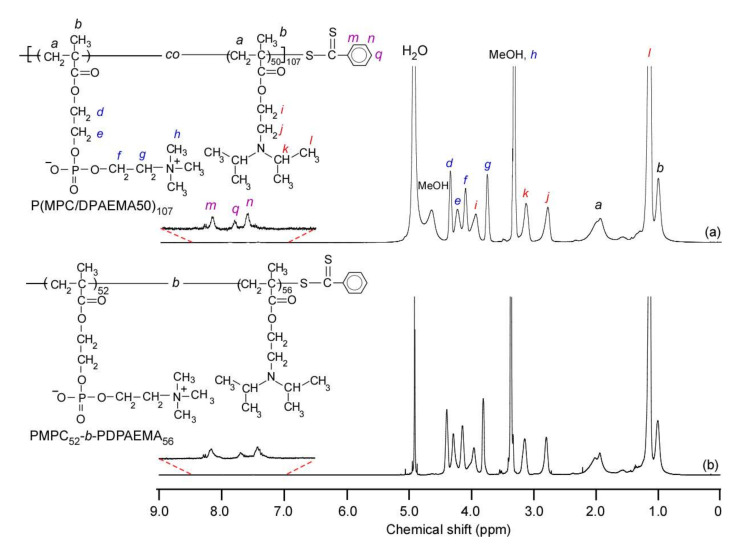
^1^H NMR spectra of (**a**) P(MPC/DPAEMA50)_107_ and (**b**) PMPC_52_-*b*-PDPAEMA_56_ in methanol-*d*_4_ at 25 °C.

Random and block copolymers were prepared via RAFT. After collecting the samples by freeze-drying, the polymer powders were dissolved in methanol-*d*_4_ to measure the corresponding ^1^H NMR spectra for confirmation of the chemical structures and estimation of the DP and composition of the random and block polymers (Figure 2). The mole percent of DPAEMA in the random copolymer was estimated to be 50 mol% using the integral intensity ratio of the methylene protons at 3.14 and 3.80 ppm derived from the DPAEMA and MPC units, respectively. The total DP value of the random copolymer was 107 according to the intensities of these two signals with the corresponding composition and the signals of the terminal phenyl groups at 7.2–8.1 ppm. The DP value of the PDPAEMA block in the block copolymer was determined to be 56 using the integral intensity ratio of the methylene protons at 3.14 ppm and the terminal phenyl groups at 7.2–8.1 ppm. The theoretical molecular weight (*M*_n_(theory)) was calculated according to the following equation:

(1) $$M_{n}\left(\mathrm{theory}\right)=\frac{{\left[M\right]}_{0}}{{\left[\mathrm{CTA}\right]}_{0}}\times \frac{p}{100}\times M_{m}+M_{\mathrm{CTA}}$$

where [M]_0_ and [CTA]_0_ are the initial concentrations of the monomers and CTA, respectively, *p* is the percentage of conversion, and *M*_m_ and *M*_CTA_ are the molecular weights of the monomer and CTA, respectively.

To determine the *M*_n_(GPC) and *M*_w_/*M*_n_ of the copolymers, GPC measurements were performed using a 0.3 M Na_2_SO_4_ aqueous solution containing 0.5 M acetic acid as an eluent (Figure S1). Narrow unimodal curves were observed with *M*_w_/*M*_n_ values of 1.03 and 1.14 for the random and block copolymers, respectively, which demonstrates that the polymerizations were well-controlled. The theoretical values of both copolymers were close to the corresponding *M*_n_(NMR) estimated using the NMR spectra. However, the *M*_n_(GPC) values were about twofold lower due to the different principles of these methods. Thus, *M*_n_(NMR) is an absolute value calculated from the intensity ratios of resonances corresponding to protons of the polymers, whereas *M*_n_(GPC) is estimated using a standard polymer sample having a different chemical structure from that of our polymer. In addition, it should be noted that although the random and block copolymers were designed to exhibit similar DPs and their *M*_n_(NMR) and *M*_n_(theory) were also similar, the *M*_n_(GPC) of the random copolymer was lower than that of the block copolymer (Table 1). This can be explained by the difference in the polymer architecture of these copolymers leading to a different interaction with the column in the GPC system, with the concomitant difference in their *M*_n_(GPC) values.

### 3.2. pH-Responsive Behavior of the Copolymers

**Figure 3:**
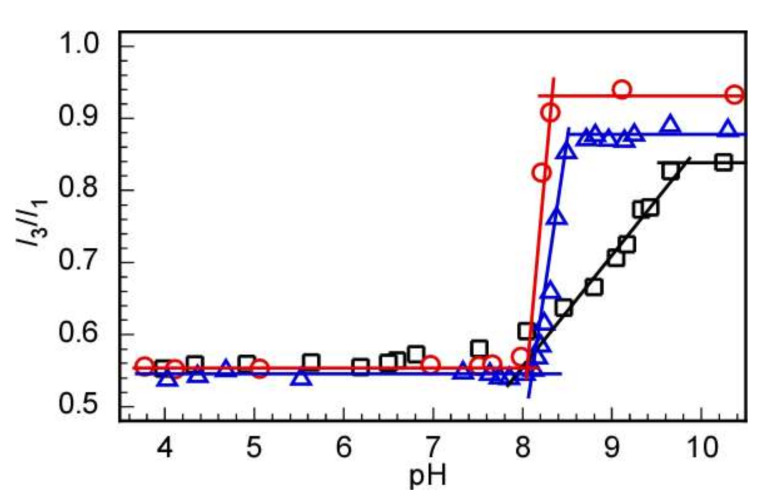
Fluorescence peak intensity ratio (*I*_3_/*I*_1_) of pyrene in the presence of (MPC/DPAEMA50)_107_ (□), PMPC_52_-*b*-PDPAEMA_56_ (ο), and their equimolar mixture (Δ) in 0.1 M NaCl at a total polymer concentration of 1.0 g/L as a function of the solution pH.

The pH-responsive behavior of the copolymers was investigated by a fluorescence probe method using Py as a hydrophobic probe, which is used as an indicator according to its surrounding polarity [30,31]. At acidic pH, the copolymers are dissolved in water as single polymer chains due to the protonation of the DPAEMA chains, and Py is directly dissolved and surrounded by water molecules. In contrast, at basic pH above the critical pH values of the copolymers, the DPAMEA units are deprotonated and DPAEMA becomes more hydrophobic, resulting in the formation of polymer micelles. In this situation, Py is entrapped in the hydrophobic core of the micelles, and its fluorescence spectrum changes accordingly, i.e., the intensity ratios between the third to the first vibronic peaks (*I*_3_/*I*_1_) increase. Therefore, aqueous solutions of the random and block copolymers and their equimolar mixture at different pH values from 3 to 11 were subjected to fluorescence measurements. The obtained *I*_3_/*I*_1_ values were plotted as a function of the solution pH to determine the critical pH values, which were found to be close to 8 for all the samples (Figure 3).

The *I*_3_/*I*_1_ of the block copolymer solution underwent a sudden change, whereas this value changed more slowly in the sample of the random copolymer. This phenomenon can be explained in terms of the different distribution of the DPAEMA units in the polymer chains in both types of copolymers. Thus, in the block copolymers, the DPAEMA units are placed close to each other in the block, which facilitates the interaction of the hydrophobic DPAEMA units when the solution pH increases slightly. However, in the case of the random copolymer, the hydrophobic interaction between hydrophobic DPAEMA units may be weaker and more difficult than that in the block polymer due to their random order along the polymer chains, resulting in a smoother change in the *I*_3_/*I*_1_ values versus the solution pH for the random copolymer.

### 3.3. Association Behavior of the Random and Block Copolymers and Their Mixture

**Figure 4:**
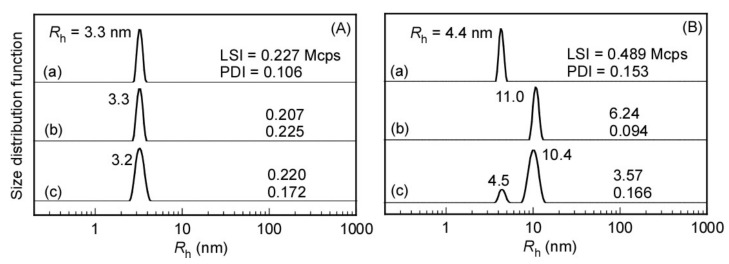
Hydrodynamic radius (*R*_h_) distributions of (**a**) P(MPC/DPAEMA50)_107_, (**b**) PMPC_52_-*b*-PDPAEMA_56_, and (**c**) an equimolar mixture of the random and block copolymers at a total polymer concentration of 10 g/L in 0.1 M NaCl at pH 3 (**A**) and 10 (**B**) at 25 °C.

DLS measurements were conducted in 0.1 M NaCl aqueous solutions at pH 3 and 10 for the random and block copolymers and their equimolar mixture (Figure 4). Before the measurements, the samples were passed through a 0.2 μm pore size filter.

At pH 3, unimodal distributions were observed in all the samples with relatively low light scattering intensity (LSI) close to 0.2 Mcps, suggesting that both copolymers were totally dissolved in water, and no attractive interaction between the random and block copolymers occurred at acidic pH. At pH 10, both *R*_h_ and LSI values increased compared with that at pH 3, which is in accord with the formation of polymer micelles in all the samples. Notably, a bimodal distribution was observed for the mixed aqueous solution of the random and block copolymers with *R*_h_ values of 4.5 and 10.4 nm (Figure 4B(c)), which are similar to those of the micelles formed from the random (Figure 4B(a)) and block copolymers (Figure 4B(b)), respectively. This suggests that separated micelles were formed in the mixture of the random and block copolymers.

**Figure 5:**
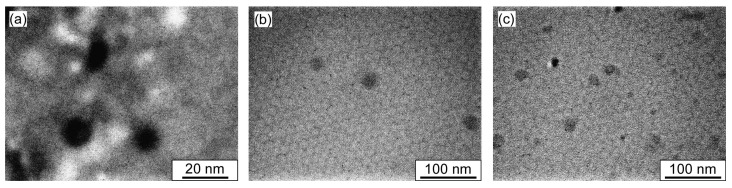
Representative transmission electron microscopy (TEM) images of (**a**) P(MPC/DPAEMA50)_107_, (**b**) PMPC_52_-*b*-PDPAEMA_56_, and (**c**) an equimolar mixture of the random and block copolymers at a total polymer concentration of 10 g/L in 0.1 M NaCl at pH 10.

The formation of the polymer micelles was confirmed by performing TEM observations, which revealed the presence of spherical particles whose sizes were in agreement with the *R*_h_ values (Figure 5 and Table 2). Particularly, two kinds of objects were observed in the TEM image of the mixture of the random and block copolymers with average sizes of 10.6 and 4.6 nm, which matches well with the results extracted from the DLS measurements.

The polymers were further characterized by performing SLS measurements (Figure S2). According to the obtained results, the *R*_g_/*R*_h_ value of the aggregates of the block copolymer was calculated to be 1.12, suggesting that the aggregates have spherical shapes [32,33,34]. In the case of the random copolymer, the *R*_g_ value could not be determined because the size of the polymer micelles was too small. The aggregation number (*N*_agg_) for the random copolymer was close to unity (0.83), which means that each single polymer chain formed unimolecular micelles in the aqueous solution. In contrast, about seven polymer chains aggregated in the polymer micelles of the block copolymer solution. These different behaviors were caused by the difference in the sequence of the hydrophobic and hydrophilic units in the polymer chains and in the balance between the hydrophilic and hydrophobic interactions. In the mixture of the random and block copolymers, the *R*_g_ value was close to that of the block copolymer micelles, whereas intermediate values between those of the random and block copolymer single solutions were obtained for *M*_w_ and *N*_agg_, which was probably caused by the coexistence of two distinct random and block copolymer micelles.

The density (*d*) of the copolymer micelles was estimated using Equation (2):

(2) $$d=\frac{M_{w}\left(\mathrm{SLS}\right)}{V\times N_{A}}\;$$

where *V* is the volume of the micelle calculated according to the expression 4/3*πR*_h_^3^, *M*_w_(SLS) is the molecular weight of the micelle estimated from the SLS measurement, and *N*_A_ is Avogadro’s number. The results are listed in Table 2. The *d* value of the aggregates produced from the block copolymers was smaller than that of the random copolymers. This implies that the block copolymer micelles were more hydrated than the random copolymers micelles, which is presumably due to the presence of the stabilizing hydrophilic PMPC blocks with a DP of 52 in the copolymers. This result is also consistent with the *R*_g_/*R*_h_ of 1.12 obtained for the block copolymer micelles. Besides, the random copolymers self-folded to form compact unimer micelles, which contributes to the higher density of this sample.

### 3.4. FRET Measurements

**Figure 6:**
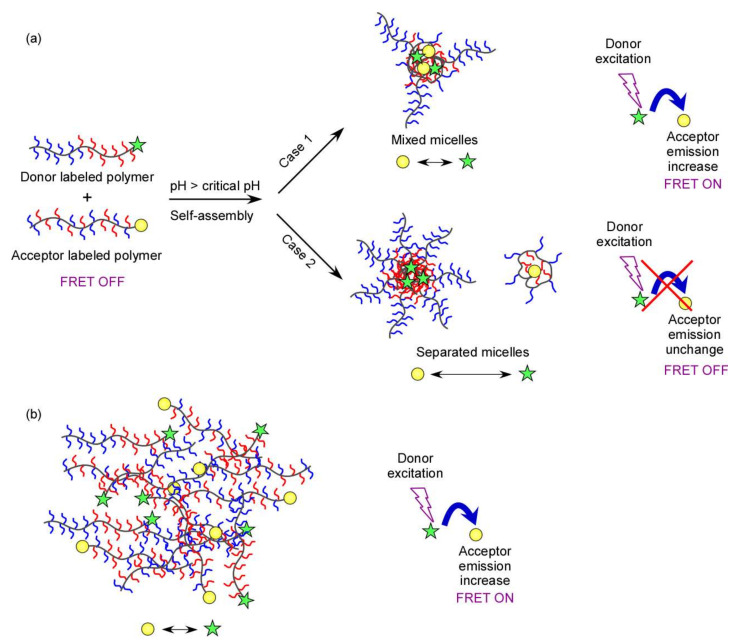
Schematic of a fluorescence resonance energy transfer (FRET) experiment in a mixture of random and block copolymers at low concentration and high concentration of donor and acceptor in (**a**) water and (**b**) a poor solvent, dimethylsulfoxide.

The terminal groups of the random and block copolymers were labeled with A555 and A488, respectively, for the FRET experiments. The chemical structures of the copolymers were confirmed to remain unchanged after labeling with the fluorescence probe on the basis of ^1^H NMR spectra recorded in D_2_O at a *C*_p_ of 10 g/L and pH 3 (Figure S3). Moreover, GPC measurements were conducted before and after labeling, which revealed that the *M*_n_ and *M*_w_/*M*_n_ of the labeled copolymers were similar to those of the copolymers before labeling (Table S1). The ^1^H NMR and GPC results prove that the structures of the copolymers were well maintained after labeling. Using the absorbance and molar extinction coefficient of A555 at 556 nm and A488 at 495 nm (Figure S4a), the content of the fluorescence label was calculated to be 8.83 and 20.8 mol% for the labeled random and block copolymers, respectively. The fluorescence emission spectra of A488 and A555 also confirmed the successful labeling (Figure S4b). These copolymers were then used for FRET experiments to investigate the formation of separated micelles in the mixture of the random and block copolymers. FRET is a process by which a fluorophore (donor) in an excited state transfers its energy to a neighboring molecule (acceptor) when donor and acceptor are close to each other. After receiving energy, the fluorophore acceptor molecule is excited and then emits to return to its ground state, and hence, its fluorescence intensity increases [35,36,37]. In a mixture of two copolymers, FRET occurs if mixed micelles are formed or the copolymers are sufficiently close. In contrast, FRET does not occur when separated micelles are formed. In our study, the random and block copolymers were independently labeled with the A555 acceptor and A488 donor, respectively. On the basis of the principle depicted in Figure 6a, at a pH above the critical pH of the solution, if mixed micelles of the random and block copolymers are formed, both donor and acceptor would locate in the same micelle (case 1). In this case, the distance between the donor and the acceptor would be smaller than the Förster radius (*R*_0_), which is the distance at which the fluorescence resonance energy transfer from the donor dye to the acceptor dye is 50% efficient. The *R*_0_ value of the A488 and A555 pair has been reported to be 7 nm [38] and the radii of the copolymer micelles obtained in our polymeric systems were found to be less than 12 nm (Table 2). Therefore, energy transfer occurs and FRET would be observed in case 1. Meanwhile, if separated micelles are formed in the mixture, the donor and acceptor would be localized in the two distinct micelles (case 2), the distance between them would exceed the *R*_0_ value, and FRET would not occur. In addition, if the labeled random and block copolymers are dissolved in a poor solvent at a sufficiently high concentration of the fluorophore for donor and acceptor to be close enough to ensure the energy transfer, highly efficient FRET could be achieved (Figure 6b).

**Figure 7:**
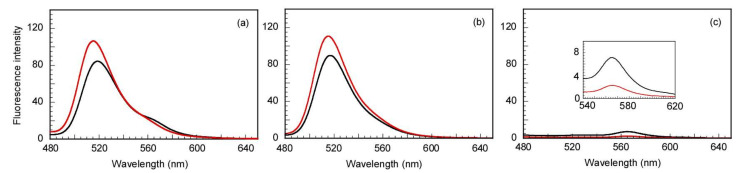
Fluorescence spectra of A488-labeled block and A555-labeled random copolymers (**a**), A488-labeled block copolymer (**b**), and A555-labeled random copolymer (**c**) in mixtures with nonlabeled random and block copolymers at a total polymer concentration of 10.0 g/L in water at pH 2 (black) and 11 (red). λex = 450 nm, the excitation and emission slit widths were 20 and 2.5 nm, respectively. A488 = Alexa Fluor 488 C5 maleimide; A555 = Alexa Fluor 555 C2 maleimide.

For these experiments, equimolar mixtures of labeled and non-labeled random and block polymers were prepared at a total *C*_p_ of 10.0 g/L, and the same concentrations of the A555 acceptor and A488 donor were used (0.030 µM). Fluorescence spectra were recorded for these samples at pH 2 and 11 (Figure 7a). The result showed that FRET did not occur at pH 11; the fluorescence intensity of the acceptor at 565 nm did not increase, indicating that the mixed micelles were not formed. However, the fluorescence intensity of donor and acceptor increased and decreased slightly, respectively, which is presumably due to their inherent properties. Therefore, to study the pH-responsive behaviors of donor and acceptor, fluorescence measurements were performed for a mixture of A488 (or A555) and nonlabeled random and block polymers (Figure 7b,c). The concentration of each polymer was the same as that used in the abovementioned FRET study. The estimation of the FRET efficiency (*E*) of the energy transfer from the donor to the acceptor can be deduced from the quenching of the donor using Equation (3):

(3) $$E=1-\frac{I_{\mathrm{DA}}}{I_{D}}$$

where $I_{D}$ and $I_{\mathrm{DA}}$ are the fluorescence intensities of the donor in the absence and presence of the acceptor at 516 nm. The fluorescence intensities and FRET efficiencies of donor and acceptor are summarized in Table S2. According to these results, upon increasing the pH, the intensity of donor and acceptor increased and decreased, respectively, in both the single solutions of each copolymer and their mixture due to their own pH-responsive behavior. Additionally, the *E* values calculated for the mixture at pH 2 and 11 were close to 0 in both cases, suggesting that there was no energy transfer from the donor to the acceptor. This suggests that separated polymer micelles were formed in the mixed aqueous solution of random and block polymers at pH 11.

**Figure 8:**
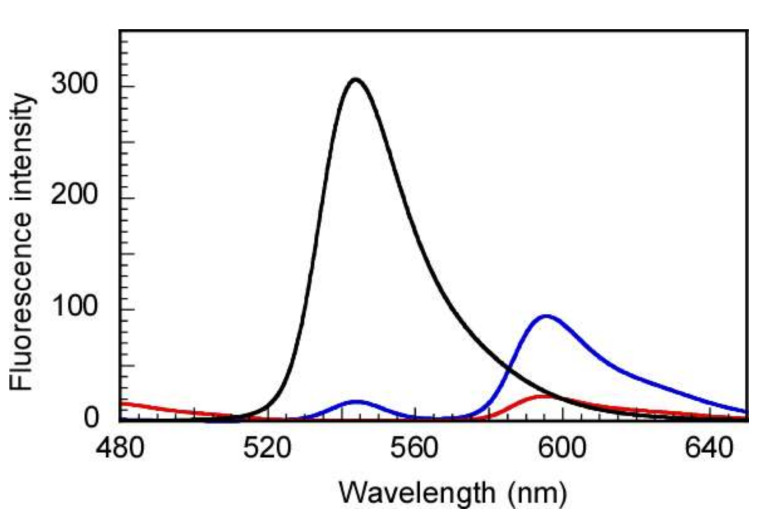
Fluorescence spectra of A488-labeled block polymer (black), A555-labeled random polymer (red), and their equimolar mixture (blue) in acidified dimethylsulfoxide; λex = 450 nm, the excitation and emission slit widths were 20 and 1.0 nm, respectively. A488 = Alexa Fluor 488 C5 maleimide; A555 = Alexa Fluor 555 C2 maleimide.

To demonstrate the efficiency of FRET in the system of A488 donor and A555 acceptor, fluorescence spectra were recorded for the single solutions of the A488-labeled block polymer, the A555-labeled random polymer, and their mixture in DMSO as a poor solvent. First, the solutions were prepared in DMSO at a *C*_p_ of 10.0 g/L for each polymer. However, the copolymers could not dissolve in DMSO, as seen with the naked eye. Thus, about 10 µL of 6 M HCl was added in 2 mL of the solutions to ensure complete dissolution of the copolymers, and the fluorescence measurements were then conducted (Figure 8). The DLS results (Figure S5) suggest that acidified DMSO is a poor solvent for both copolymers because bimodal distributions were observed for the random and block copolymers and their mixture with a relatively high *R*_h_ value, which indicates that the copolymers were not completely dissolved. For this reason, when the labeled copolymers solutions were prepared in acidified DMSO with a high *C*_p_ of 10.0 g/L, the random and block labeled copolymers were in a crowded distribution, and the distance between donor and acceptor was appropriate for FRET. Theoretically, the FRET efficiency would be maximized when the higher proportion of the donor molecule that transfers excitation energy to the acceptor molecules is obtained. In this situation, the *I*_DA_ value would decrease, and the *E* value would be close to 1. In the mixture of the random and block copolymers in acidified DMSO, the *E* value was calculated to be 0.943, indicating that FRET occurred with high efficiency. This observation indicates that the A488 donor and A555 acceptor worked well.

The FRET experiments clearly demonstrate that separated micelles were formed in the mixture of the random and block copolymers composed of MPC and DPAEMA. This phenomenon might be due to the incompatibility of these copolymers caused by the different arrangement of the monomer units along the polymer chains. Moreover, Py fluorescence spectra were recorded in the presence and absence of the random or block copolymers in 0.1 M NaCl at different *C*_p_ to determine the critical micelle concentration (CMC) values of these copolymers. Py is an indicator for the hydrophobicity of its surrounding environment [30,31]. Below CMC, the fluorescent intensity ratio (*I*_3_/*I*_1_) between the third to the first peak in the Py emission spectra is low, and this value increases when the copolymer micelles formed at a *C*_p_ higher than CMC. Using this, *I*_3_/*I*_1_ values were plotted as a function of the copolymer concentration to estimate the CMC (Figure S6). CMC value of the block copolymer was determined to be 0.0055 g/L. When *C*_p_ increased to be around or higher than CMC, *I*_3_/*I*_1_ gradually increased because Py was entrapped in the hydrophobic core of the micelles. Whereas, in the mixture of Py and the random copolymer, *I*_3_/*I*_1_ values were nearly the same when *C*_p_ varied from 0.0005 to 0.05 g/L, indicating that unimer micelles always formed regardless of the *C*_p_. Hydrophobicity of the hydrophobic domain of the unimer micelle is lower than that of the block copolymer micelles.

## 4. Conclusions

Random and block copolymers were successfully prepared from MPC and DPAEMA via RAFT polymerization. In aqueous environment, the critical pH values of the random and block copolymers and their equimolar mixture were close to 8. These copolymers self-assembled to form polymer micelles in aqueous solutions at pH values above the critical pH. Separated micelles were formed in the mixture of these copolymers, which indicated that the compatibility in the structure of the copolymers is important for their assembly. The formation of separated micelles was confirmed by the appearance of a bimodal size distribution in their mixed aqueous solution and by the absence of FRET between the acceptor and donor in the random and block copolymer chains. The sizes of the polymer aggregates were less than 100 nm, which renders them as promising carriers in drug delivery systems.

## Figures and Tables

**Table 1:** Number-Average Molecular Weight (*M*_n_), and Molecular Weight Distribution (*M*_w_*/M*_n_) of P(MPC/DPAEMA50)_107_ and PMPC_52_-*b*-PDPAMEA_56_.

Sample	*M*_n_(theo) *^a^* × 10^−4^ (g/mol)	*M*_n_(NMR) *^b^* × 10^−4^ (g/mol)	*M*_n_(GPC) *^c^* × 10^−4^ (g/mol)	*M*_w_ */M*_n_ *^c^*
P(MPC/DPAEMA50)_107_	2.51	2.75	1.02	1.03
PMPC_52_-*b*-PDPAMEA_56_	2.53	2.76	1.49	1.14

*^a^* Calculated from Equation (1). *^b^* Calculated from DP(NMR), which was estimated according to the ^1^H NMR spectra using the integrated intensity ratio of pendant methylene and terminal phenyl protons and the molecular weights of monomer and CTA with the corresponding compositions. *^c^* Estimated via GPC measurements.

**Table 2:** Characteristics of the Random and Block Copolymers and Their Equimolar Mixture Micelles in 0.1 M NaCl at pH 10 and 25 °C.

Sample	d*n*/d*C*_p_	*R*_h_*^a^* (nm)	*R*_TEM_*^b^* (nm)	*R*_g_*^c^* (nm)	*R*_g_/*R*_h_	*M*_w_*^c^* × 10^5^ (g/mol)	*N*_agg_ *^d^*	*d ^e^* (g/cm^3^)
Random	0.253	4.4	5.2	-	-	0.23	0.83	0.108
Block	0.274	11.0	12.7	12.3	1.12	2.11	6.7	0.063
Mixture	0.228	10.4 (4.5) *^f^*	10.6 (4.6) *^g^*	12.2	-	1.21	4.6	-

*^a^* Determined via DLS analysis. *^b^* Estimated from TEM images. *^c^* Molecular weights of the polymer micelles estimated from SLS measurements. *^d^* Aggregation number (*N*_agg_) values, the number of individual polymer chains incorporated in one micelle, were calculated using *M*_w_ *^c^*/(*M*_n_ × *M*_w_/*M*_n_). *M*_n_ and *M*_w_/*M*_n_ are the number-average molecular weight and molecular weight distribution of the individual polymer chains estimated from NMR and GPC measurements, respectively. In the case of the mixture of random and block copolymers, the average *M*_n_ value was estimated from the SLS measurement at pH = 3. *^e^* Density calculated using Equation (2). *^f^* Bimodal *R*_h_ distributions. *^g^* Two different sizes were observed.

## Data Availability

The data presented in this study are available in this study. Additional information could be available on request from the corresponding author.

## Supplementary Materials

The following supporting information can be downloaded at: https://www.mdpi.com/article/10.3390/polym14030577/s1, Scheme S1. Synthesis of (a) random copolymer P(MPC/DPAEMA50)_107_ and (b) block copolymer PMPC_52_-*b*-PDPAEMA_56_, Scheme S2. Elimination of the RAFT end groups and labeling of (a) PMPC_52_-*b*-PDPAEMA_56_ with Alexa Fluor 488 C_5_ maleimide (A488) and (b) P(MPC/DPAEMA50)_107_ with Alexa Fluor 555 C_2_ maleimide (A555), Figure S1. Gel-permetion chromatography (GPC) elution curves of (a) P(MPC/DPAEMA50)_107_ and (b) PMPC_52_-*b*-PDPAEMA_56_ using a 0.3 M Na_2_SO_4_ aqueous solution containing 0.5 M acetic acid as an eluent at 40 °C, Figure S2. Zimm plots for (a) P(MPC/DPAEMA50)_107_ and (b) PMPC_52_-*b*-PDPAEMA_56_ at a polymer concentration (*C*_p_) of 2.0 g/L at pH 10 and (c) an equimolar mixture of the random and block copolymers at a *C*_p_ of 5.0 g/L at pH 10 in 0.1 M NaCl at 25 °C, Figure S3. ^1^H NMR spectra of PMPC_52_-*b*-PDPAEMA_56_ (a) before and (b) after labeling with Alexa Fluor 488 C_5_ maleimide and P(MPC/DPAEMA50)_107_ (c) before and (d) after labeling with Alexa Fluor 555 C_2_ maleimide in D_2_O at pH 3 and 25 °C, Table S1. Characterization of P(MPC/DPAEMA50)_107_ and PMPC_52_-*b*-PDPAEMA_56_ Before and After Fluorescence-labeling, Figure S4. (a) UV–vis absorption and (b) fluorescence spectra of A555-labeled P(MPC/DPAEMA50)_107_ (λ_ex_ = 520 nm) (—) and A488-labeled PMPC_52_-*b*-PDPAEMA_56_ (λ_ex_ = 493 nm) (—) in pure water at 25 °C. A488 = Alexa Fluor 488 C_5_ maleimide; A555 = Alexa Fluor 555 C_2_ maleimide, Table S2. Fluorescence Intensities of Donor and Acceptor, Figure S5. Hydrodynamic radius (*R*_h_) distributions of (a) P(MPC/DPAEMA50)_107_, (b) PMPC_52_-*b*-PDPAEMA_56_, and (c) an equimolar mixture of the random and block copolymers in acidified dimethylsulfoxide at 25 °C. Figure S6. Fluorescence peak intensity ratio (*I*_3_/*I*_1_) of pyrene in the presence of (MPC/DPAEMA50)_107_ (□) and PMPC_52_-*b*-PDPAEMA_56_ (ο) in 0.1 M NaCl plotted against the polymer concentration (*C*_p_). *I*_3_ and *I*_1_ are the fluorescence intensities of the third and the first vibronic peaks, respectively, in the pyrene emission spectra recorded at the excitation wavelength of 334 nm.
