# Supplementary material for: Separated Micelles Formation of pH-Responsive Random and Block Copolymers Containing Phosphorylcholine Groups
Source: Polymers (Basel). 2022 Jan 31;14(3):577. doi: 10.3390/polym14030577 (PMC8838922; doi:10.3390/polym14030577)
Supplement: Supplementary file 1 [file polymers-14-00577-s001.zip › polymers-1553920-supplementary.pdf]

Supporting information

Separated Micelles Formation of pH-Responsive Random and Block Copolymers Containing Phosphorylcholine Groups

Thi Lien Nguyen[1], Kazuhiko Ishihara[2] and Shin-ichi Yusa[1,*]

[1] Department of Applied Chemistry, Graduate School of Engineering, University of Hyogo, 2167 Shosha, Himeji, Hyogo 671-2280, Japan; nguyenlienk56hh@gmail.com

[2] Department of Materials Engineering, The University of Tokyo, 7-3-1 Hongo, Bunkyo-ku, Tokyo 113-8656, Japan; ishihara@mpc.t.u-tokyo.ac.jp

* Correspondence: yusa@eng.u-hyogo.ac.jp

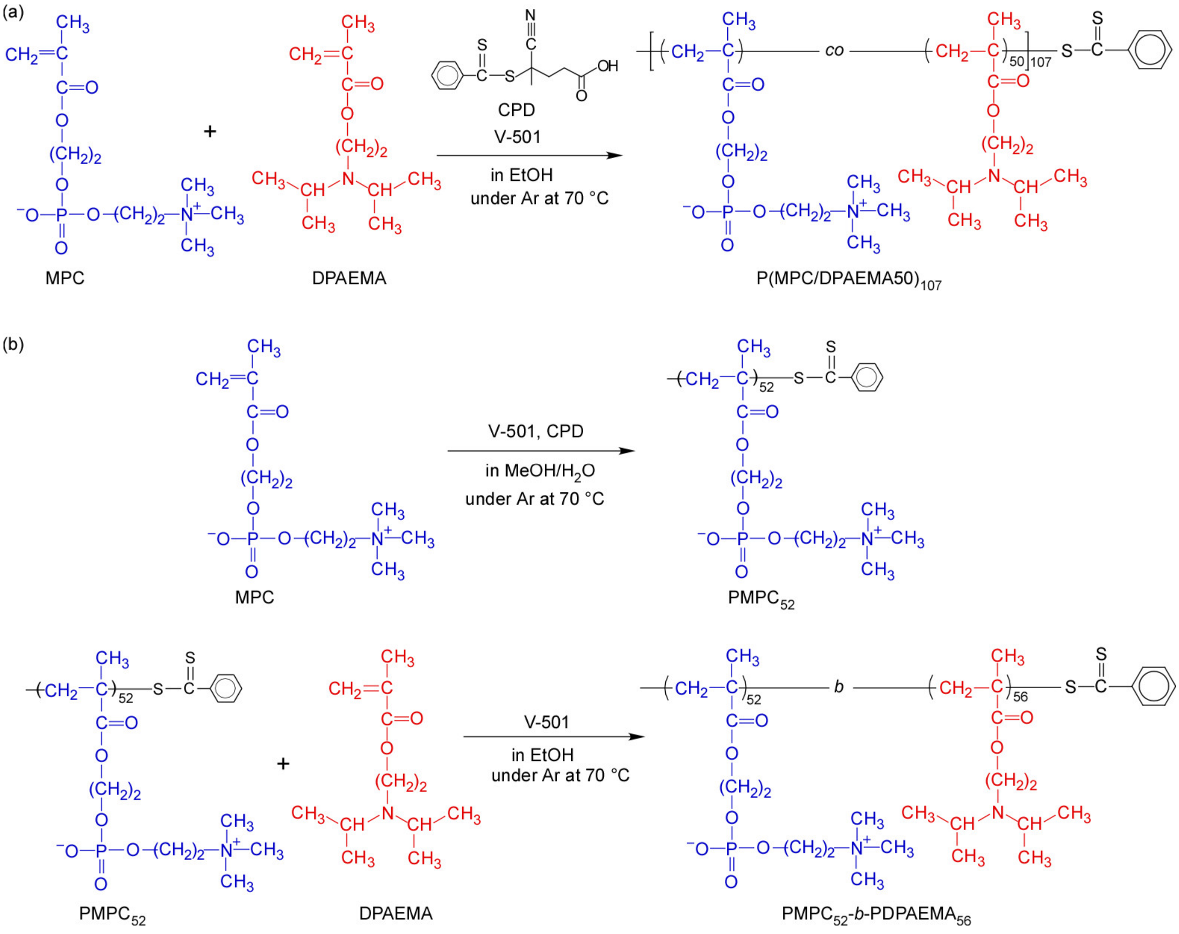

Scheme S1. Synthesis of (a) random copolymer P(MPC/DPAEMA50)$_{107}$ and (b) block copolymer PMPC$_{52}$-*b*-PDPAEMA$_{56}$.

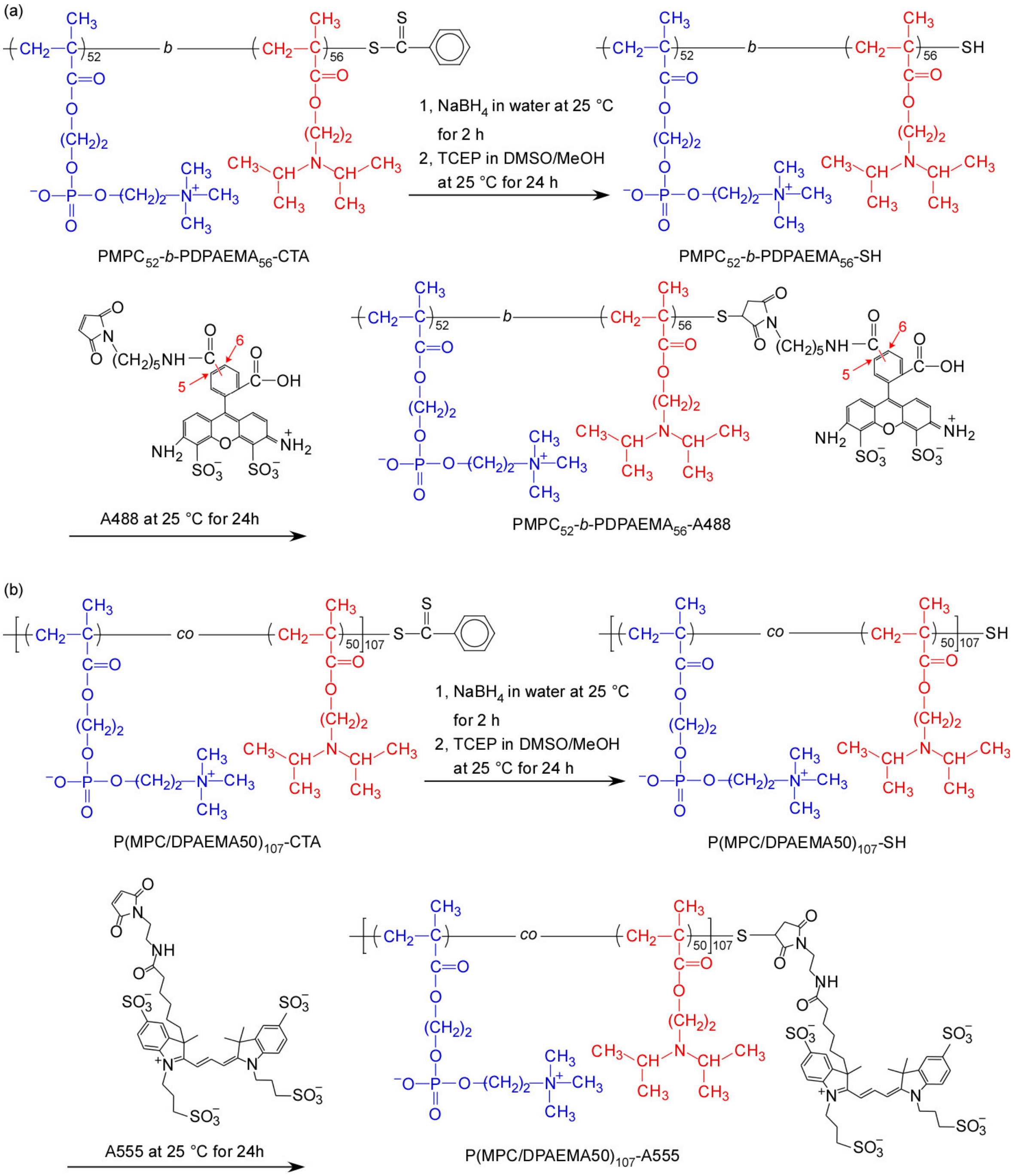

Scheme S2. Elimination of the RAFT end groups and labeling of (a) $PMPC_{52}$-*b*-$PDPAEMA_{56}$ with Alexa Fluor 488 C_5 maleimide (A488) and (b) $P(MPC/DPAEMA50)_{107}$ with Alexa Fluor 555 C_2 maleimide (A555).

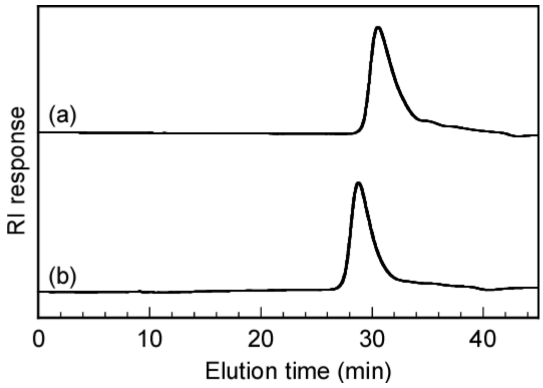

Figure S1. Gel-permetion chromatography (GPC) elution curves of (a) P(MPC/DPAEMA50)$_{107}$ and (b) PMPC$_{52}$-*b*-PDPAEMA$_{56}$ using a 0.3 M Na_2SO_4 aqueous solution containing 0.5 M acetic acid as an eluent at 40°C.

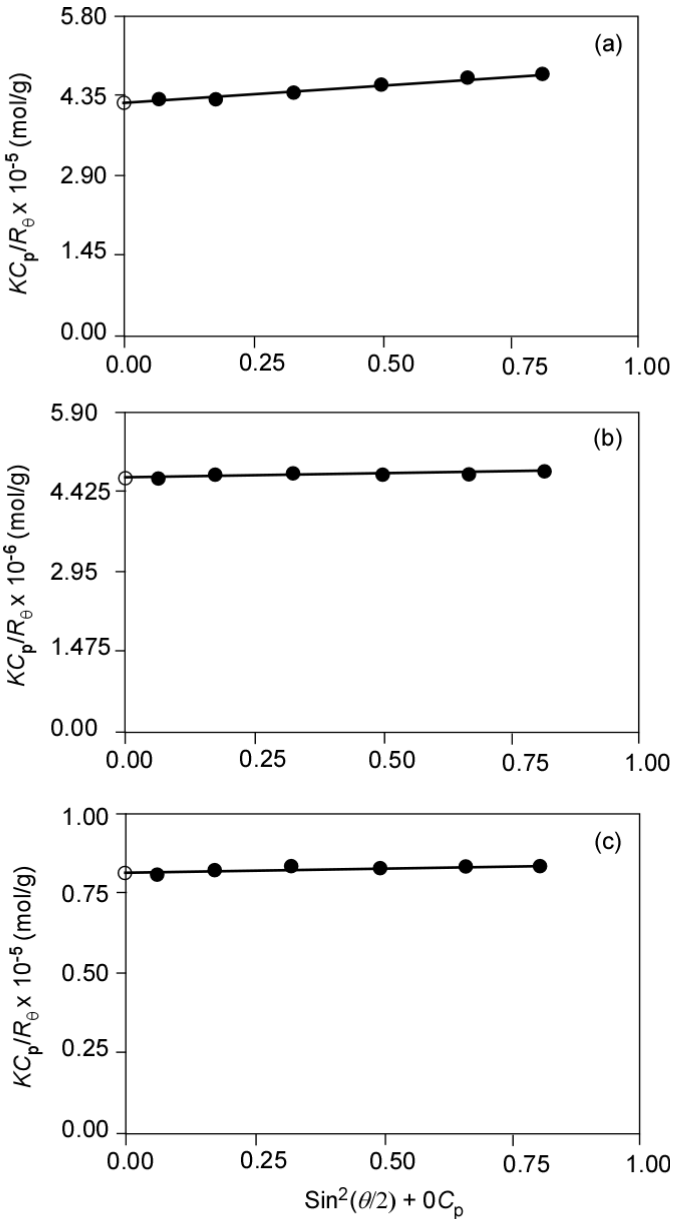

Figure S2. Zimm plots for (a) P(MPC/DPAEMA50)$_{107}$ and (b) PMPC$_{52}$-*b*-PDPAEMA$_{56}$ at a polymer concentration (C_p) of 2.0 g/L at pH 10 and (c) an equimolar mixture of the random and block copolymers at a C_p of 5.0 g/L at pH 10 in 0.1 M NaCl at 25°C.

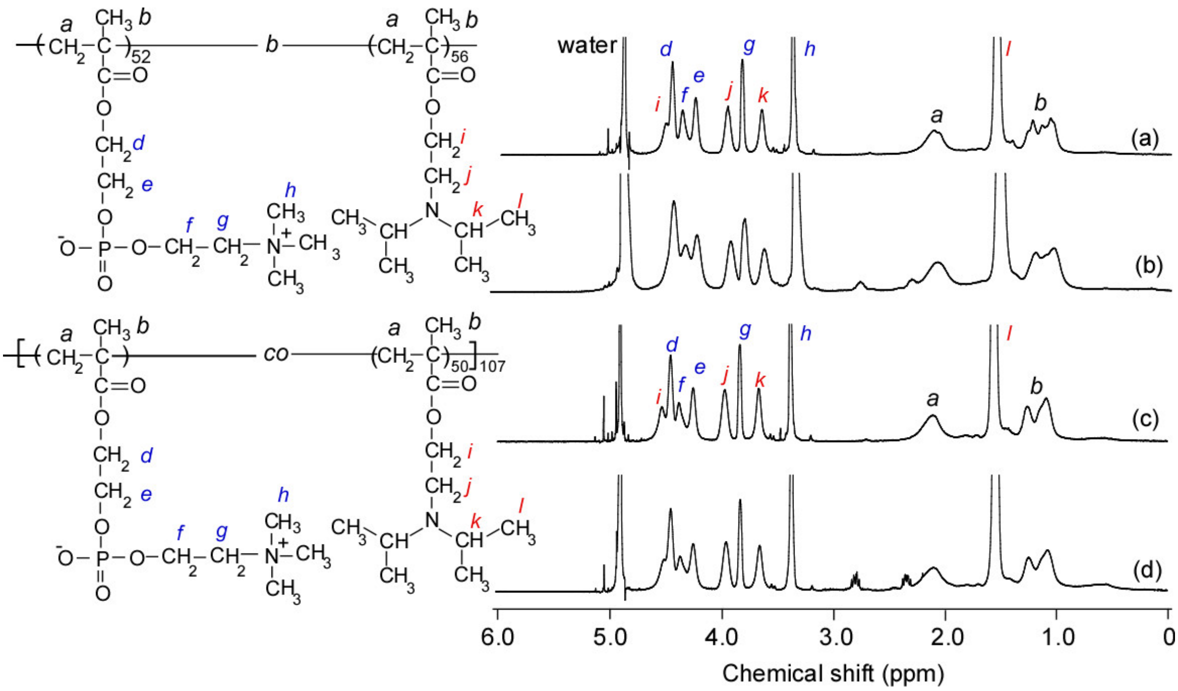

Figure S3. ^{1}H NMR spectra of $PMPC_{52}$-*b*-$PDPAEMA_{56}$ (a) before and (b) after labeling with Alexa Fluor 488 C_5 maleimide and $P(MPC/DPAEMA50)_{107}$ (c) before and (d) after labeling withAlexa Fluor 555 C_2 maleimide in D_2O at pH 3 and 25°C.

Table S1. Characterization of $P(MPC/DPAEMA50)_{107}$ and $PMPC_{52}$-*b*-$PDPAEMA_{56}$ Before and After Fluorescence-labeling

	$P(MPC/DPAEMA50)_{107}$		$PMPC_{52}$-*b*-$PDPAEMA_{56}$	
	Before labeling	After labeling by A555	Before labeling	After labeling by A488
$M_n \times 10^{-4}$ (g/mol)	1.02	1.19	1.49	1.62
M_w/M_n	1.03	1.44	1.14	1.36

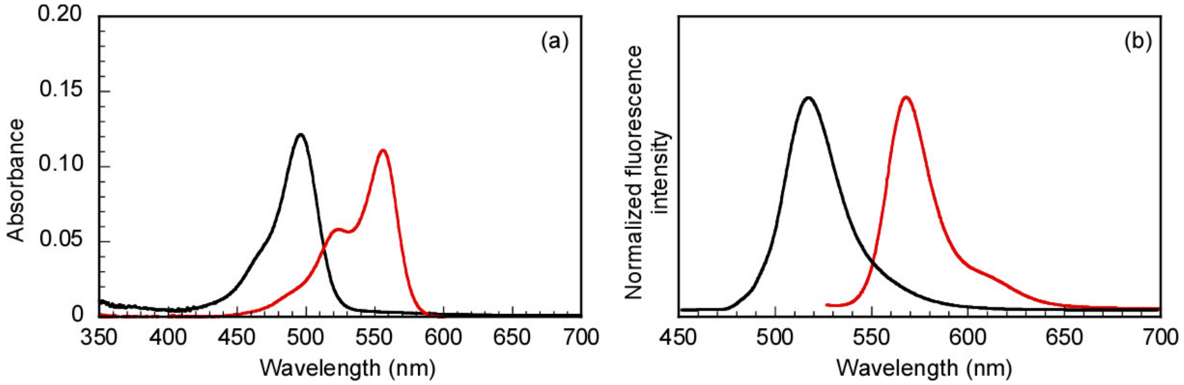

Figure S4. (a) UV–vis absorption and (b) fluorescence spectra of A555-labeled P(MPC/DPAEMA50)$_{107}$ (λ_{ex} = 520 nm) (—) and A488-labeled PMPC$_{52}$-*b*-PDPAEMA$_{56}$ (λ_{ex} = 493 nm) (—) in pure water at 25°C. A488 = Alexa Fluor 488 C$_5$ maleimide; A555 = Alexa Fluor 555 C$_2$ maleimide.

Table S2. Fluorescence Intensities of Donor and Acceptor

Sample	Fluorescence intensity				E^c	
	$I_D{}^a$		$I_A{}^b$			
	pH 2	pH 11	pH 2	pH 11	pH 2	pH 11
Mixture of donor and acceptor	82.6	106.2	8.63	2.87	0.0768	0.0389
Donor only	89.5	110.5	-	-	-	-
Acceptor only	-	-	7.16	2.29	-	-

[a]Fluorescence intensity of the donor (A488 at 516 nm). I_D = 0 in the solution of the acceptor only. [b]Fluorescence intensity of the acceptor (A555 at 565 nm). I_A = 0 in the solution of the donor only. [c]FRET efficiency calculated using the expression $1 - I_{DA}/I_D$, where I_D and I_{DA} are the fluorescence intensities of the donor in the absence and presence of the acceptor at 516 nm.

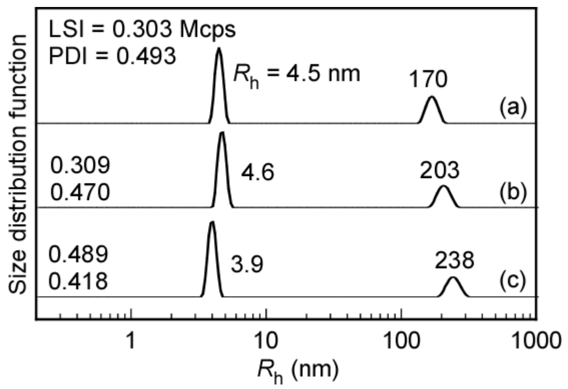

Figure S5. Hydrodynamic radius (R_h) distributions of (a) P(MPC/DPAEMA50)$_{107}$, (b) PMPC$_{52}$-*b*-PDPAEMA$_{56}$, and (c) an equimolar mixture of the random and block copolymers in acidified dimethylsulfoxide at 25°C.

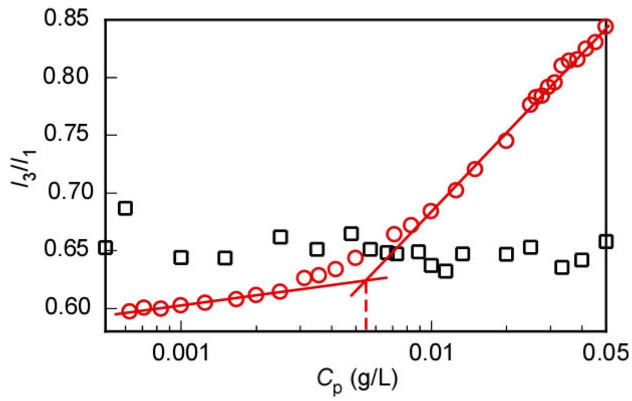

Figure S6. Fluorescence peak intensity ratio (I_3/I_1) of pyrene in the presence of (MPC/DPAEMA50)$_{107}$ (□) and PMPC$_{52}$-*b*-PDPAEMA$_{56}$ (○) in 0.1 M NaCl plotted against the polymer concentration (C_p). I_3 and I_1 are the fluorescence intensities of the third and the first vibronic peaks, respectively, in the pyrene emission spectra recorded at the excitation wavelength of 334 nm.
